# Bacillaceae Within *Galium aparine* L.: A Plant‐Associated Treasure of Biosynthetic Diversity and Growth‐Promoting Potential

**DOI:** 10.1111/1758-2229.70447

**Published:** 2026-10-23

**Authors:** Natalia Rutkowska, Piotr Drożdżyński, Tomasz Boruta, Bartosz Sekuła, Olga Marchut‐Mikołajczyk

**Affiliations:** ^1^ Institute of Molecular and Industrial Biotechnology, Faculty of Biotechnology and Food Sciences Lodz University of Technology Lodz Poland; ^2^ Department of Bioprocess Engineering Faculty of Process and Environmental Engineering, Lodz University of Technology Lodz Poland

**Keywords:** abiotic stress, Bacillaceae, endophytic bacteria, *Galium aparine* L, plant growth, plant microbiome

## Abstract

Endophytic bacteria are key components of plant microbiomes, enhancing host growth, stress tolerance, and defence mechanisms. Ten cultivable strains affiliated with Bacillaceae, including putative novel *Priestia* and *Bacillus* species, were previously isolated from *Galium aparine* L.'s tissues. All tolerated diverse levels of abiotic stresses, produced ammonia and auxins, as well as all exhibited strong hydrolytic enzyme activities (amylase, protease, xylanase, and pectinase), indicating robust metabolic versatility. These bacteria stand out as prolific producers of industrially relevant compounds. *Priestia megaterium* GR4 demonstrated high production of hydrolytic enzymes and biosurfactants, and most strains synthesized and accumulated polyhydroxyalkanoates (PHA), highlighting their potential as sustainable bioplastic producers. UPLC‐MS/MS confirmed koranimine production by *Peribacillus frigoritolerans* GR2. Previous genome mining studies revealed diverse biosynthetic gene clusters, including those for surfactin and fengycin. Overall, *G. aparine* endophytes represent powerful biological factories capable of producing a wide range of valuable compounds, including enzymes, biosurfactants and biopolymers, with strong potential for applications in industrial biotechnology, sustainable agriculture, and biocontrol.

## Graphical abstract

The study involved 10 strains of endophytic bacteria isolated for the first time from *G. aparine* L., including two putatively novel strains from *Bacillus* and *Priestia* spp. Bioactivity screening revealed production of a plethora of industrially‐relevant compounds and possible use of those bacteria as biostimulants.

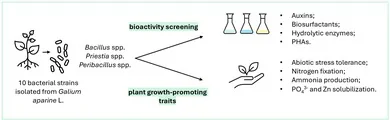

**Abbreviations**
BGCs: biosynthetic gene clusters
CAZymes: carbohydrate‐active enzymes
CCD: central composite design
CMC: carboxymethylcellulose
EMFs: electromagnetic fields
GluCl: glutamate‐gated chloride channel
HMMs: Hidden Markov Models
IAA: indole‐3‐acetic acid
IAAld: indole‐3‐acetaldehyde
IAM: indole‐3‐acetamide
IAN: indole‐3‐acetonitrile
IPA: indole‐3‐pyruvate
LB: Luria‐Bertani
LSs: lyophilized supernatants
MIBiG: Minimum Information about a Biosynthetic Gene cluster
NB: nutrient broth
NCBI: National Center for Biotechnology Information
NRPs: non‐ribosomally synthesized peptides
OFAT: one factor at the time
PEG: polyethylene glycol
PGAP: prokaryotic genome annotation pipeline
PHA: polyhydroxyalkanoates
PHB: poly‐β‐hydroxybutyrate
PKs: polyketides
PWD: pine wilt disease
RiPPs: ribosomally synthesized and post‐translationally modified peptides
RSM: response of the surface methodology
SB staining: Sudan Black B staining
SD: standard deviation
SDS: sodium dodecyl sulfate
TPM: tryptamine
TSB: trypton‐soyton broth
UPLC‐MS: ultra performance liquid chromatography tandem mass spectrometry

## 1 Introduction

During many years of co‐existence, plants have developed close relationships with the surrounding microorganisms. Those microorganisms help them thrive in changing environments, facilitating nutrient acquisition and protection from both biotic and abiotic stress. The most peculiar group of those microorganisms are endophytes (mostly bacteria and fungi) living inside their tissues without causing any harm to their host (Afzal et al. 2019; Eid et al. 2021). Endophytes do not necessarily spend their entire life cycle inside plants, but at least a portion of it. They can enter from micropores, hydathodes, or wounds, or even be transmitted by seeds or bulbs through the plants' offspring (Drożdżyński et al. 2024).

Endophytic microorganisms have long been known for their beneficial properties for their host. The main ones include enhancement of nutrient acquisition (nitrogen, phosphorus, and iron), production of phytohormones supporting growth or production of defence metabolites (antibiotics, metallophores, and hydrolytic enzymes) (Eid et al. 2021). Although not every plant has been studied to this point, it is assumed that all of them contain endophytes, without which are essential to the plant survival (Partida‐Martínez and Heil 2011). Interestingly, some endophytes—especially those transmitted through plant offspring—can produce the same or highly similar bioactive compounds of medicinal use as their plant host. This makes it possible for them to be used as an industrial supply of those compounds, more sustainable than extraction from plants (Kusari and Spiteller 2011; Rutkowska et al. 2023).

Among the most prevailing phyla of the culturable endophytic bacteria in diverse plants are Firmicutes with *Bacillus* genus at the forefront (Lopes et al. 2018). Members of the genus *Bacillus*, such as *B. thuringiensis* , *B. velezensis* , *B. cereus* , and *B. subtilis* have been long recognized as valuable plant partners. They are known for conferring resistance against insect pests, nematodes, and environmental stresses, and also for the production of hydrolytic enzymes, siderophores, biosurfactants, and antimicrobials (Andrić et al. 2023; Lalitha and Nithyapriya 2021; Marchut‐Mikołajczyk et al. 2023; Palma et al. 2014). Because of their spore‐forming ability, *Bacillus* strains are readily adaptable to commercial formulation and field applications as safe alternatives to current crop management practices (Lopes et al. 2018; Radhakrishnan et al. 2017).

Identification of biotechnologically applicable compounds produced by *Bacillus* species so far has relied on labor‐intensive screening methods and biochemical characterizations. However, the use of these methods often results in a high rediscovery rate (Scherlach and Hertweck 2021). Recent advances in sequencing technology expanded the number of available *Bacillus* species' genome assemblies in the NCBI GenBank database (www.ncbi.nlm.nih.gov/genbank/), with almost 16,000 genomes deposited up to August 2025. Acquired genomic data enables broad *in silico* analysis of putative genes responsible for bioactive compounds production (Blin et al. 2023; Yin et al. 2023). The major products of their secondary metabolism include ribosomally synthesized and post‐translationally modified peptides (RiPPs), non‐ribosomally synthesized peptides (NRPs) and polyketides (PKs) (Dilshad et al. 2022; Rutledge and Challis 2015). The genes responsible for synthesizing these compounds are typically organized in close proximity within biosynthetic gene clusters (BGCs). The so‐called genome mining approach encompasses searching through the genome for conserved sequence motifs or protein domains based on known sequences of BGC, for example, using statistical models as Hidden Markov Models (HMMs) (Blin et al. 2023). Discovering gene clusters from whole genome sequence enables exploration of the full potential of endophytic *Bacillus* spp. species as an underexploited source of novel molecules of biotechnological interest.

*Galium aparine* L. is a worldwide‐occurring annual herbaceous plant. Herbaceous plants hold immense economic importance due to the production of compounds applicable in the pharmaceutical industry (Drożdżyński et al. 2024). *G. aparine* (cleavers) is known for its beneficial effects on kidneys, skin disorders, wounds, high blood pressure, and insomnia (Al‐Snafi 2018). In folk medicine, it was used as a pulp to treat venomous bites and stings, as well as a cold or hot brewed tea. The fruits of *G. aparine* L. contain caffeine (the plant itself belongs to the same family as coffee), thus are used as a stimulant substitute for it (Al‐Snafi 2018; Ilina et al. 2019). Apparently, no studies about its association with endophytes have been conducted before. In 2024, we isolated 10 bacterial strains from roots, stem, and leaves of *G. aparine* L. plant growing in the middle of Poland, and studied their genomes in detail for the presence of genes involved in plant growth, enzyme metabolism, and production of secondary metabolites (Rutkowska et al. 2025).

This study analysed endophytic bacteria isolated from *G. aparine* L. for their tolerance to stress conditions, including heat, drought, and salinity, traits that enhance direct growth promotion, as well as their capacity to produce hydrolytic enzymes, phytohormones, siderophores, and surface‐active compounds, predicted through genome mining.

## 2 Results and Discussion

Our most recent study provides the first comprehensive insight into the cultivable bacterial community associated with the medicinal plant *G. aparine* L. (Rutkowska et al. 2025). Ten bacterial strains were isolated from different tissues of surface‐sterilized *G. aparine* L. and subjected to *de novo* genome assembly using both short‐ and long‐read sequencing techniques. Whole‐genome‐based taxonomic analysis revealed that all isolates belong to the family Bacillaceae, with two isolates identified as *Priestia* sp. GS2 and *Bacillus* sp. GL1, both potentially representing novel species as they show low similarity to any known species. As part of this investigation, we analysed the genomes for the presence of genes associated with plant growth promotion, carbohydrate‐active enzymes (CAZymes), and BGCs. All genomic data have been deposited in the NCBI database under BioProject accession number PRJNA1068863.

### 2.1 Abiotic Stress Tolerance

Profiting from the genetic insights obtained from the Bacillaceae isolates associated with *G. aparine* , we assessed their physiological responses and plant growth‐promoting potential under various abiotic stress conditions. All isolates were evaluated for their ability to grow under varying temperature, salinity, and drought stress conditions (Table 1). Each isolate demonstrated growth within a temperature range of 20°C–45°C and tolerated NaCl concentrations ranging from 0.5% to 5%. Notably, several isolates—*Bacillus pretiosus* GR1, *Bacillus thuringiensis* GS1, *Bacillus* sp. GL1, *Bacillus cereus* GL2 and *Bacillus wiedmannii* GL3—were capable of growing at 10°C. In addition, *Bacillus pretiosus* GR1, *Priestia megaterium* GR4, *Priestia* sp. GS2, *B. cereus* GS3, and *B. cereus* GL2 exhibited tolerance to high salinity, sustaining growth at 10% NaCl. Also, all isolates showed resilience to water‐deficit stress induced by the addition of 10% and 20% polyethylene glycol (PEG) 8000 to the broth medium. In a similar study, *Bacillus* spp. strains isolated from desert *Lepidium perfoliatum* were similarly capable of growing in media containing 20% PEG 6000, with the majority also tolerating 30% PEG (Li et al. 2017). Furthermore, 89% of those isolates grew in the presence of 12% NaCl, and 95% maintained growth at 45°C, indicating a high level of environmental tolerance. Establishing symbiotic relationships with such stress‐tolerant microorganisms, particularly endophytes, can significantly enhance plant resilience under abiotic stress conditions. The plants in the present study were collected from a small, forested area near a military airport in Łask, Poland. The impact of the airports on wildlife is still underestimated; however, light pollution is defined as the most significant. Light pollution primarily affects birds, but it also delays flowering and developmental patterns in plants. Noise, vibration and electromagnetic fields (EMFs) effects on plants are highly understudied (Radomska et al. 2021). Available data suggest that EMFs directly interfere with plant responses and their tolerance to stress, primarily by decreasing photosynthetic efficiency. Additionally, vibration stress can reduce plant height due to a decrease in the modulus of elasticity and bending force (Far et al. 2019; Tran et al. 2023). There are also indications that suboptimal levels of EMFs can increase plant biomass production, revealing its prospects for sustainable agriculture (Ayesha et al. 2023). Studies show that sound stimulation can actually trigger the production of secondary metabolites, which are necessary for further enhancement of seed germination, flowering, growth, and defence (Wu et al. 2023). Endophytic bacteria as crucial plant partners also contribute to their response to various stressors.

**TABLE 1:** Environmental tolerance limits for isolates.

Strain	Temperature (°C)					NaCl (%)				PEG 8000 (%)		
	10	20	30	45	55	0.5	5	10	15	0	10	20
*Bacillus pretiosus* GR1	+	+	+	+	−	+	+	+	−	+	+	+
*Peribacillus frigoritolerans* GR2	−	+	+	+	−	+	+	−	−	+	+	+
*Bacillus cereus* GR3	−	+	+	+	−	+	+	−	−	+	+	+
*Priestia megaterium* GR4	−	+	+	+	−	+	+	+	−	+	+	+
*Bacillus thuringiensis* GS1	+	+	+	+	−	+	+	−	−	+	+	+
*Priestia* sp. GS2	−	+	+	+	−	+	+	+	−	+	+	+
*Bacillus cereus* GS3	−	+	+	+	−	+	+	+	−	+	+	+
*Bacillus* sp. GL1	+	+	+	+	−	+	+	−	−	+	+	+
*Bacillus cereus* GL2	+	+	+	+	−	+	+	+	−	+	+	+
*Bacillus wiedmannii* GL3	+	+	+	+	−	+	+	−	−	+	+	+

For instance, *Bacillus halotolerans* LBG‐1‐13 isolated from roots of lily seedlings demonstrated tolerance to high salinity (10% NaCl) and osmotic stress (20% PEG 8000) in the growth medium. It also exhibited multiple plant growth‐promoting traits, such as auxin production, phosphate solubilization and activities against fungal phytopathogens (Gao et al. 2022).

### 2.2 Plant Growth‐Promoting Traits

Direct plant growth‐promoting activities of the *G. aparine* L. strains were assessed as preliminary screening tests. All 10 strains showed ammonia production under the conditions tested; however, none exhibited phosphate or zinc solubilization. Ammonia production by endophytes confers resistance in plants against pathogens and directly promotes growth through a sustained nitrogen supply (Afzal et al. 2019). Several strains, precisely *Peribacillus frigoritolerans* GR2, *B. cereus* GS3, *B. cereus* GL2, and *Priestia* sp. GS2, were able to fixate nitrogen. These observations should be considered indicative and preliminary, as they are based solely on phenotypic assays and do not constitute definitive evidence of metabolic capability.

Auxins, represented by indole‐3‐acetic acid (IAA), are key phytohormones in plant development, involved in cell enlargement and division, tissue differentiation and many other physiological processes. Plant‐associated bacteria are often able to produce IAA on their own and therefore modulate the level of plant IAA to optimal or supraoptimal levels (Etesami and Glick 2024). Their synthesis depends on the type of pathway, dependent or independent of the presence of tryptophan (precursor) (Zhang et al. 2019; Zhao 2010). The production of IAA can vary greatly between species and strains within the same genus, influenced by environmental conditions, growth rates and availability of carbon and nitrogen sources (Etesami and Glick 2024).

Our results showed that all of the isolated endophytic strains were able to produce IAA phytohormone in the presence of 0.2% (w/v) L‐tryptophan, IAA precursor (Figure 1, Table S1).

**FIGURE 1:**
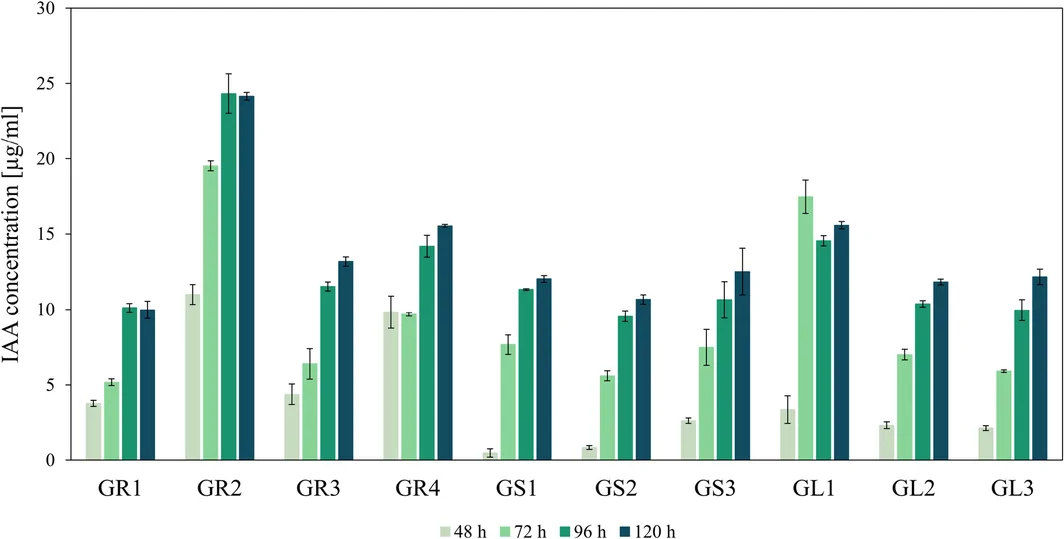
IAA production (μg/ml) by *Galium aparine* L.'s bacterial isolates. Error bars represent standard deviation values.

The production rate at 96 h ranged from 9.98 μg/mL by *B. pretiosus* GR1 to 24.33 μg/mL by *P. frigoritolerans* GR2; however, the production in all other species oscillates at a level of approximately 12–15 μg/mL for 96 and 120 h. For most isolates, production increased gradually and was the highest after 120 h of cultivation, which suggests that the enzymes transforming tryptophan to IAA are still active, and IAA is accumulating. Tryptophan is converted to IAA by different pathways, with four of them clarified to some extent, named according to their main intermediate: indole‐3‐acetonitrile (IAN), indole‐3‐acetamide (IAM), indole‐3‐pyruvate (IPA), and tryptamine (TPM). In genomes of all *G. aparine* L.'s isolates, we found genes involved in tryptophan biosynthesis, as well as a gene encoding indole‐3‐pyruvate decarboxylase enzyme (EC 4.1.1.74) in strains belonging to the *B. cereus* group (GS1, GS3, GL1, GL2, GL3, and GR3) (Rutkowska et al. 2025). Indole‐3‐pyruvate decarboxylase catalyses the conversion of indole pyruvate (IPA) to indole‐3‐acetaldehyde (IAAld), suggesting that most likely the IAA production in these strains occurs according to the IPA pathway. Results obtained via large‐scale genomic analysis conducted by Zhang et al. (2019) suggest that Firmicutes genomes have the simplest Trp‐dependent IAA synthetic systems and a plethora of bacteria could potentially synthesize IAA via multiple incomplete pathways (Zhang et al. 2019).

Numerous plant‐associated bacteria were found to be producers of IAA (Etesami and Glick 2024; Wagi and Ahmed 2019). *P. frigoritolerans* 2RO30 isolated from *Brassica napus* produced 22.57 ± 1.11 μg/mL in medium with 100 μg/L L‐Trp after 96 h of cultivation, which is comparable to our *P. frigoritolerans* GR2, which generated 24.33 μg/mL (Świątczak et al. 2024). *Priestia megaterium* BP‐R2 isolated from the halophyte plant *Bolboschoenus planiculmis* in LB medium supplemented with 100 μg/L L‐tryptophan yielded up to 31 μg/mL IAA after 48 h (Hwang et al. 2022). Inoculation of *Arabidopsis thaliana* and *Brassica rapa* L. plants with that strain significantly improved many growth characteristics of the host plants under normal and salt and drought stress conditions compared to those of the mock‐inoculated plants (Hwang et al. 2022). Another endophytic isolate, taken from *Vigna radiata* , including *Priestia aryabhattai* MBN3, *P. megaterium* MJHN10, and *B. cereus* MJHN1 displayed production of significant amounts of IAA after only an initial screening in YEM medium supplemented with 100 μgg/L L‐Trp—92.03 ± 1.2, 71.33 ± 1.0, and 68.27 ± 0.5 μg/mL, respectively, after 5 days (Bhutani et al. 2018). Also, further optimization of L‐Trp concentration and nitrogen and carbon sources resulted in higher yields, reaching up to 150 μg/mL. On the contrary, the remaining 30 tested strains isolated from the same source, which remain unidentified, produced significantly smaller quantities (Bhutani et al. 2018). Another study emphasizing the crucial role of optimization described rhizospheric *B. cereus* So3II isolated from *Solanum nigrum* and *Bacillus subtilis* Mt3b from *Macrosiagon tricuspidatum*, in which optimum IAA producing potential was almost 10 folds higher than that from the initial screening (241.6 μg/mL from 35.8 μg/mL for So3II, 322.6 μg/mL from 36.6 μg/mL for Mt3b) (Wagi and Ahmed 2019). Interestingly, both strains could produce IAA without the addition of precursor; however, less, which suggests the presence of at least two complementary pathways. Genomic research of *B. thuringiensis* RZ2MS9 isolated from guarana rhizosphere showed that it harbours the complete set of genes required in two pathways of IAA production—indole‐3‐pyruvate (IPA) and tryptamine (TPM); however, it produced relatively small amount of IAA (max. 0.20 μg/mL; 1 g/L L‐Trp) (Batista et al. 2021). Zhang et al. (2019), indicated that several genomes may harbour up to three distinct yet intact pathways or four various incomplete pathways simultaneously (Zhang et al. 2019). Along with optimization, bacterial cell immobilization presents another method for enhancing IAA production. Özdal et al. (2016) studied four rhizospheric *B. cereus* strains for IAA production. The BC‐On strain was found to be the best IAA producer with 46.25 μg/mL after 72 h of cultivation. Subsequent immobilization led to an IAA yield of 300 μg/mL after only 18 h of fermentation. Immobilized cells in alginate beads were repeatedly used in 13 successive reaction cycles (each cycle 18 h) without a decline in IAA yield (Özdal et al. 2016).

### 2.3 Hydrolytic Potential

The ability of the tested bacterial endophytes isolated from *G. aparine* L. to produce hydrolytic enzymes involved in degradation of cellulose, starch, proteins, fats, chitin, chitosan, xylan, pectin and lignin was tested on LB agar medium with addition of appropriate substrates. Summary of the results is presented in Table 2, examples of activity are in Figure 2A–D.

**TABLE 2:** Enzymatic activities of bacterial endophytes isolated from *Galium aparine* L.

Enzymes	Strains									
	GR1	GR2	GR3	GR4	GS1	GS2	GS3	GL1	GL2	GL3
Cellulases	−	−	+	+++	+	−	++	−	++	+++
Amylases	+++	+	++	+++	+	+++	++	++	++	++
Proteases	++	+	+	+++	+	+++	++	+	+	++
Esterases	−	++	+	++	−	+	+	+	+	−
Lipases	−	−	−	−	−	−	−	−	−	−
Chitinases	++	−	−	−	++	−	+	−	+	+
Chitosanases	−	+	++	+	+	−	+	+	+	+
Xylanases	+++	+	+	+++	+	++	++	+	++	++
Pectinases	+++	++	+	+++	+	++	+	++	+	+
Laccases	−	−	−	+++	−	+	−	−	−	−

*Note:* ‘−’—lack of growth/activity/halos; ‘+’—activity present/slight halos (1‐2 mm); ‘++’—medium halos (3–4 mm); ‘+++’—large halos (≥ 5 mm).

**FIGURE 2:**
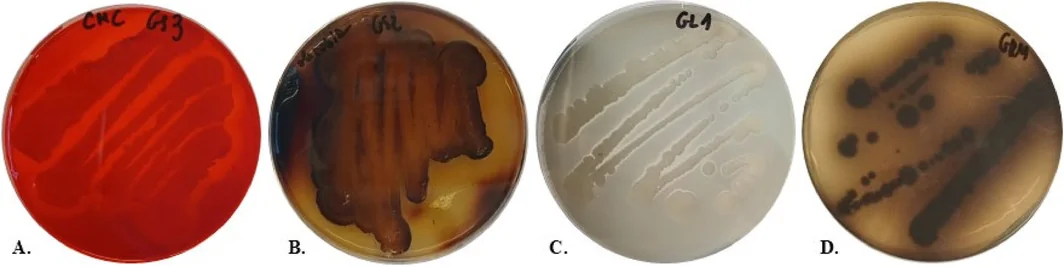
Examples of enzymatic activities of *Galium aparine* L.'s bacterial endophytes. (A) Cellulases— *Bacillus cereus* GS3, (B) amylases—*Priestia* sp. GS2, (C) lipases—*Bacillus* sp. GL1, and (D) laccases—*P. megaterium* GR4.

All of the tested bacteria demonstrated amylolytic and proteolytic activity, which is a characteristic of *Bacillus* spp. Additionally, nearly all strains displayed slight esterolytic activity, while none displayed lipolytic activity. Esterolytic activity refers to ester bonds in general, whereas lipolytic activity is a specific type of degrading lipid substrates. Furthermore, all strains exhibited xylanolytic and pectinolytic activity, some much higher than others, including *B. pretiosus* GR2 and *P. megaterium* GR4. However, only *P. megaterium* GR4 showed strong potential for lignin degradation. *Priestia* sp. GS2 demonstrated a slight level of such activity. In our previous research, we analysed carbohydrate‐active enzymes (CAZymes) encoded within the genomes of endophytes associated with *G. aparine* L. (Rutkowska et al. 2025). *Priestia* sp. GS2 and *P. megaterium* GR4 have the highest percentage of CAZymes to the total gene number, 2.87% and 2.42%, respectively, with a greater number of glycoside hydrolases than other isolated strains. This is reflected by the test results on agar plates (Figure 2A–D). It should be noted that the extracellular enzyme activities reported in this study were assessed using qualitative plate‐based screening assays. The results should be interpreted as preliminary indicators of enzymatic capability rather than absolute enzymatic rates. Further quantitative assays would be required to accurately determine enzyme kinetics and activity levels under defined conditions.

Production of hydrolytic enzymes enables endophytic bacteria to enter plant tissues through the hydrolysis of cell wall components; thus allowing them to inhabit the interior and support proper growth and yield (Eid et al. 2021). Members of *Bacillus* spp. are very profound producers of hydrolytic enzymes. In the study of endophytic bacteria isolated from *V. radiata* and *Cajanus cajan* plants, nine isolates out of 47 demonstrated the ability to produce amylases, cellulases, proteases, and pectinases, and five of them were assigned to *Bacillus* genus (Bhutani et al. 2021). All strains also demonstrated moderate IAA and ammonia production, some phosphate solubilization and siderophore production, which are all important plant‐growth promoting traits.

The pot experiment demonstrated a notable enhancement in all measured plant growth parameters when compared to the uninoculated control seeds, indicating that endophytic bacteria may serve as beneficial alternatives to harmful agrochemicals in agricultural practices. Similarly, *Bacillus licheniformis* BaDB06 and *Bacillus velezensis* SM‐95 from medicinal *Lessertia frutescens* , which showed the production of amylases, proteases and lipases, have proven themselves as effective bio‐agents (Tsipinana et al. 2024). Inoculation of wheat seedlings with endophytic *Bacillus* and *Paenibacillus* spp. from medicinal plant *Lonicera japonica* showed a positive correlation between enzyme (cellulase and pectinase) activity and inhibition of phytopathogenic *Fusarium oxysporum*, possibly due to production of such hydrolytic enzymes (Zhao et al. 2015). Numerous studies back this claim; for instance, *Vicia faba* ‐associated *Bacillus* spp. strains showed potential to reduce another pathogen *Fusarium equiseti* wilt in Fava bean and improve its growth (Haddoudi et al. 2021). A total of 71% of the subjects simultaneously produced cellulases, amylases and proteases, while 21% additionally produced the fourth one—pectinase. These hydrolytic enzymes, including chitinases, chitosanases, but also proteases, lipases and cellulases, due to the complexity of fungi cell wall components, have potential applications in the biological control of phytopathogens and insect pests in forestry and fruit tree production (Ajuna et al. 2023).

There is a strong interest in microbial enzymes due to their commercial applications in various fields, including the textile industry, food processing, agricultural research, and pharmaceuticals. Still, their high production cost poses one of the main challenges to the widespread use of enzymes in these industries (Singh et al. 2016). Optimizing both enzyme production and microbial growth is crucial for the economic feasibility of the whole process. This optimization requires studying various parameters involved in enzyme biosynthesis, such as media composition, pH and temperature. Statistical tools are particularly useful for this purpose. Nawawi et al. (2022) used response of the surface methodology (RSM) with a central composite design (CCD) to optimize xylanase and pectinase production by *Bacillus amyloliquefaciens* ADI2 from agro‐industrial waste carbon sources, such as f.e. oil palm trunk, orange peel, apple pomace or corn stalks (Nawawi et al. 2022). Optimum composition and conditions resulted in the production of 159.08 U/mL of xylanase and 204.86 U/mL of pectinase. That CCD model demonstrated a 1.34‐fold and 5.96‐fold increase in the production of xylanase and pectinase, respectively, compared the one factor at the time (OFAT) approach. Yadav et al. (2020) tested a bit more extreme approach (Yadav et al. 2020). They used low‐frequency ultrasonic radiation, which increased cell wall permeability and enhanced nutrient uptake, resulting in improved yields of pectinase, cellulase, and xylanase by *B. subtilis* ABDR01.

### 2.4 Production of Bioactive Compounds

#### 2.4.1 Biosurfactants

Biosurfactants possess various applications in agriculture, such as enhancing soil quality and facilitating plant growth. They can also augment the biodegradation of contaminants due to their degradative activities. Biosurfactants can substitute the harsh synthetic surfactants utilized in the pesticide industry, as they serve as carbon sources for soil‐dwelling bacteria, facilitating their removal from the soil. Table 3 presents the evaluation of bacterial isolates regarding their capacity to produce biosurfactants, along with their basic structural characteristics and properties.

**TABLE 3:** Characterization of biosurfactant‐producing potential of *Galium aparine* L.'s isolates.

Strain	Emulsifying activity OD_500_	Emulsion index (%)	Oil displacement test^a^	Carbohydrates concentration (μg/mL)^b^	Protein concentration (μg/mL)^b^
GR1	2.92 ± 0.05	47.29 ± 3.68	++++	7.13 ± 0.14	59.39 ± 0.45
GR2	2.92 ± 0.06	5.35 ± 0.14	++	0.28 ± 0.01	34.38 ± 0.41
GR3	3.17 ± 0.06	38.04 ± 6.47	++++	15.71 ± 0.23	11.16 ± 0.60
GR4	3.44 ± 0.03	48.96 ± 1.02	+++++	0.19 ± 0.01	33.41 ± 0.74
GS1	1.81 ± 0.07	24.64 ± 1.01	+	22.81 ± 0.08	37.76 ± 1.09
GS2	1.15 ± 0.14	11.88 ± 0.29	++	28.19 ± 0.39	41.18 ± 3.02
GS3	1.57 ± 0.08	12.59 ± 0.31	−	79.55 ± 0.36	19.25 ± 0.90
GL1	1.93 ± 0.04	10.46 ± 0.23	++++	0.17 ± 0.02	37.42 ± 1.28
GL2	3.02 ± 0.08	38.87 ± 4.46	++++	0.17 ± 0.02	29.27 ± 0.45
GL3	2.05 ± 0.07	30.17 ± 0.72	++++	0.26 ± 0.01	34.68 ± 0.63

^a^ Results of oil displacement test are expressed on the basis of speed and extent of oil displacement immediately after application the sample onto the oil surface. The symbol ‘+++++’ indicated immediate and extensive oil displacement, while ‘+’ indicated minimal oil dispersion with barely visible surface disruption; symbol ‘−’ indicated lack of response of the sample on the surface of the oil.

^b^ Data is represented by means and standard deviations values.

The strains isolated from the plant stem, named *B. thuringiensis* GS1, *Priestia* sp. GS2, and *B. cereus* GS3, demonstrated a modest degree of emulsifying activity, with OD_500_ values between 1.15 and 1.81. Conversely, the emulsifying activity of the other strains exceeded an OD_500_ of 3— *B. cereus* GR3, *P. megaterium* GR4, and *B. cereus* GL2.

It is frequently stated that the genus *Bacillus* produces biosurfactants with high emulsifying activity and surface tension decrease. These microbes typically create lipopeptides, although some strains also make glycolipids. Marchut‐Mikolajczyk et al. (2018) reported significantly lower values in their research on endophytic bacterial strains extracted from *Chelidonium majus* L., with emulsifying activity ranging from 0.23 to 1.96, the peak emulsifying activity (1.96) being attributed to the *Bacillus pumilus* 2A strain (Marchut‐Mikolajczyk et al. 2018). The investigation into the biosurfactant production capabilities of endophytic strains from *G. aparine* L. revealed that strain GR4 had the maximum emulsifying activity, measured at 3.44 ± 0.03. The emulsion index values varied from slightly above 5% to nearly 50%. The peak result was recorded for strain GR4 at 48.96% ± 1.02%. The result is analogous to the findings of Baoune et al. (2018) regarding endophytic *Streptomyces* spp. isolated from a plant in Algeria, where the emulsion index did not surpass 46% (Baoune et al. 2018). Furthermore, this result is also comparable to that reported by Marchut‐Mikolajczyk et al. (2018), which indicated the highest emulsion index for the *B. cereus* EN18 strain at 47.11% ± 3.86% (Marchut‐Mikolajczyk et al. 2018). These results indicate that strains are capable of forming stable emulsions.

The oil displacement test revealed considerable variability in the surface activity among the analysed isolates indicating the differences in the biosurfactant production ability. Sample assigned with the highest score ‘+++++’ (GR4 strain) caused immediate and extensive dispersion of the oil layer, suggesting strong surface‐active properties. In contrast, samples of GR2, GS1, GS2 and GS3 showed weak or even a lack of oil displacement activity, suggesting a limited biosurfactant production. The oil displacement assay directly reflects the production of extracellular surface‐active agents; therefore, the results obtained confirm the biosurfactant‐producing potential of six isolates.

Analysis of carbohydrate and protein concentrations in the studied bacterial strains provided key information on the likely biochemical composition of their biosurfactants, particularly concerning their emulsifying and surface‐active properties. Among the strains isolated from roots, *B. pretiosus* GR1 and *Paenibacillus megaterium* GR4 stand out for their high emulsifying activity, significant emulsion index, and strong oil‐displacing abilities. Importantly, both strains exhibited a biosurfactant profile dominated by proteins rather than carbohydrates. The sample obtained from the GR1 strain exhibited a protein concentration of 59.39 μg/mL and a moderate carbohydrate content of 7.13 μg/mL, while the sample from GR4 contained a similarly high protein concentration of 33.41 μg/mL and an unusually low carbohydrate level of 0.19 μg/mL. This protein‐rich composition, combined with their pronounced surface activity, suggests that the biosurfactants produced by these strains are likely lipopeptides.

On the other hand, other strains, such as GR3, exhibit a distinct profile characterized by reduced protein content (11.16 μg/mL) and relatively elevated carbohydrate levels (15.71 μg/mL), suggesting the possible synthesis of glycolipid or glycoprotein biosurfactants. GR3 possesses strong emulsifying and oil‐displacing properties; however, its macromolecular composition indicates a unique class of biosurfactants in contrast to GR1 and GR4. Sakr et al. obtained similar results using lactic acid bacteria to produce biosurfactants. From 81 isolates, they screened 12 that exhibited potential ability for biosurfactant production. Their results suggest *Lactobacillus plantarum* 60FHE strain was the best biosurfactant producer—glycoprotein with a high emulsion index of 50.0% ± 0.23%, oil displacement, and foaming properties (Sakr et al. 2021).

Additional heterogeneity was noted among strains GS1, GS2 and especially GS3, which exhibit carbohydrate‐dominant profiles. GS3, despite its elevated carbohydrate concentration (79.55 μg/mL), lacks oil‐displacing activity, suggesting that its extracellular products may be polysaccharides and have limited surface‐active properties. Strains GL1, GL2 and GL3 are characterized by a consistent low carbohydrate (0.17–0.26 μg/mL) and moderate protein (29.27–37.42 μg/mL) profile, as well as significant oil‐displacing activity. This supports the hypothesis that protein‐based biosurfactants, likely lipopeptides, are responsible for their surface activity. These results highlight the promise of root‐derived strains, particularly GR4, for synthesizing effective biosurfactants for applications requiring enhanced surface activity.

Analysis of the BGCs in all investigated strains revealed that *Priestia* sp. GS2 is the sole strain that lacks an evident BGC linked to biosurfactant production (Rutkowska et al. 2025). In contrast, another member of the *Priestia* genus, *P. megaterium* GR4, emerged as the most promising lipopeptide producer, possessing a BGC homologous to the surfactin biosynthetic gene cluster found in *B. velezensis* FZB42, which is deposited in Minimum Information about a Biosynthetic Gene cluster (MIBiG) database under BGC0000433. All remaining strains, classified within *Bacillus* spp., possess a BGC similar to the fengycin BGC also described in *B. velezensis* FZB42 (MIBiG no. BGC0001095). Both surfactin and fengycin are cyclic lipopeptides synthesized nonribosomally via nonribosomal peptide synthetases (NRPSs), and exhibit antibacterial and antifungal activities, respectively (Jasim et al. 2016; Li et al. 2019).

#### 2.4.2 Pigments

*Bacillus* and related species can produce pigments, often carotenoids (Khaneja et al. 2010). Both *Priestia* spp. strains (GS2, GR4) were producing yellowish colonies on agar media. Knowing that in their genomic sequences there are clusters similar to those responsible for carotenoid production in *Halobacillus halophilus* DSM 2266 (MIBiG no. BGC0000645), the production of such pigments was tested in liquid medium. Both pigments were extracted, resuspended in methanol, and subjected to UV–Vis spectrum measurement (Figure 3A).

**FIGURE 3:**
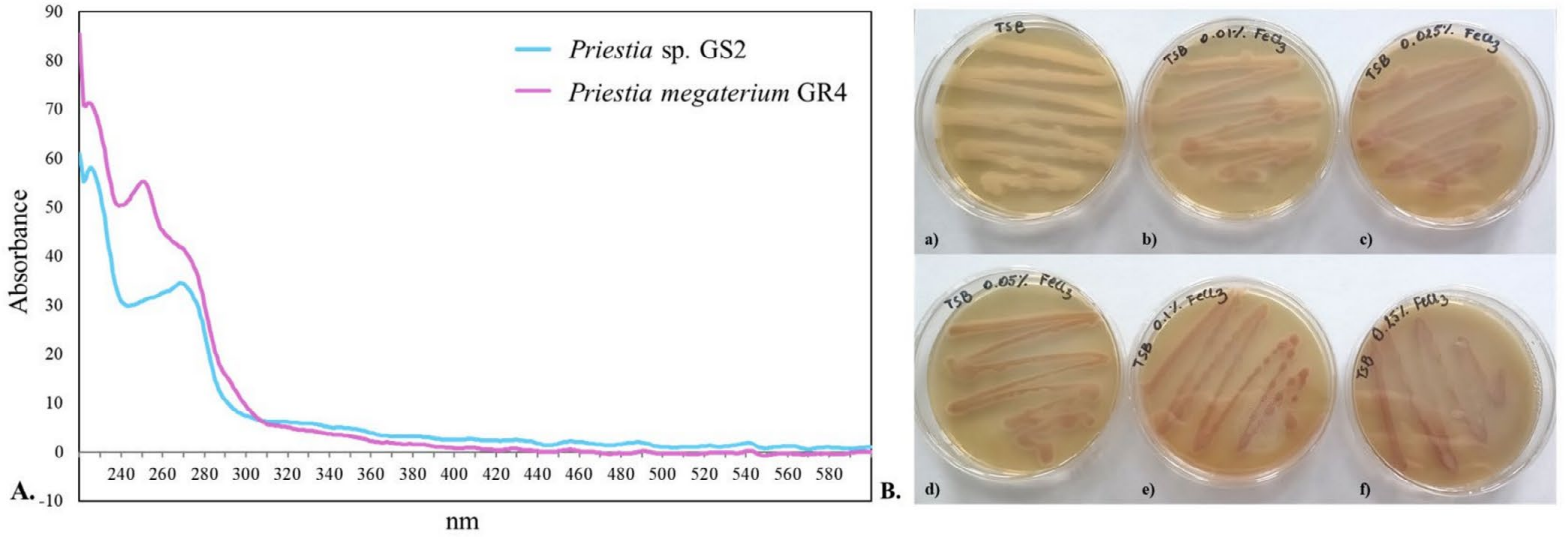
Pigment characterization and the effect of iron supplementation on pigment production by selected bacterial strains. (A) UV–Vis spectra of pigments produced by *Priestia* sp. GS2 and *Priestia megaterium* GR4. (B) Production of red pigment by *Bacillus wiedmannii* GL3 on TSB agar medium supplemented with different concentrations of FeCl_3_: (a) 0%, (b) 0.01%, (c) 0.025%, (d) 0.05%, (e) 0.1%, and (f) 0.25%.

The spectrum of pigment from *P. megaterium* GR4 shows two peaks at approximately 250 nm and 270 nm, while *Priestia* sp. GS2 has a single peak at 270 nm. The observed peaks indicate that the extracted pigments are not carotenoids, as these compounds generally display absorption peaks within the visible spectrum, specifically between 400 and 550 nm (Scott 2001). However, literature suggests the possibility of carotenoid precursors, such as apo‐8′‐phytoene isomers and menaquinone‐7, which display *λ*_max_ in the range of 240–330 nm (Perez‐Fons et al. 2011). Another potential compound is riboflavin (vitamin B2), produced by plants and most microorganisms, which has maximum absorption peaks at 224, 268, 370 and 445 nm; however, not all of these peaks are visible in our spectrum (de Jesus et al. 2012). Microbial pigments are gaining popularity as they provide a safer alternative to harmful synthetic dyes. Their antioxidant and antimicrobial properties make them suitable for applications in the food and cosmetic industries (Khaneja et al. 2010). Currently, the production of pigments relies heavily on chemical synthesis and plant extraction methods, both of which are unsustainable and environmentally harmful (Ram et al. 2020).

Genome mining of *B. wiedmannii* GL3 indicated the potential for pulcherriminic acid production due to the presence of the yvmC‐cypX operon (Fernandez and Simmons 2024; Rutkowska et al. 2025). Thus, various FeCl_3_ concentrations on TSB agar media were tested to evaluate pulcherrimin production (Figure 3B). Red pigment, likely pulcherrimin, formed inside and around bacterial colonies, while the iron content was increasing.

Pulcherriminic acid is derived from cyclic dipeptide *L*‐Leu‐*L*‐Leu, and can chelate four Fe^3+^ ions, leading to the formation of reddish pigment pulcherrimin. The best known producers are yeasts, especially *Meschnikowia pulcherrima* and *Candida pulcherrima* (Kluyver et al. 1953; Shomi et al. 2021); however, some *B. subtilis* strains are also reported to be pulcherrimin producers (Angelini et al. 2023). Charron‐Lamoureux et al. (2023) studied the role of pulcherrimin using two bacterial species model, common rhizosphere inhabitants— *B. subtilis* and *Pseudomonas* spp. They found that *B. subtilis* produces pulcherrimin in interaction with *P. protegens* Pf‐5 and creates a local source of precipitated Fe^3+^ from which it can take iron by strong siderophore bacillibactin (Charron‐Lamoureux et al. 2023). The concentration of iron in the natural environment is relatively low, which gives microorganisms that can store iron as pulcherrimin a competitive advantage. Pulcherrimin has strong antimicrobial activity against postharvest and human pathogens, antioxidant and photoprotective properties (Kregiel et al. 2022). Thus, its application in the food, agricultural, and medical industries appears promising. Microorganisms that produce significant amounts of pulcherrimin are of considerable interest to biotechnologists.

### 2.5 Other Bioactive Metabolites

#### 2.5.1 Polyhydroxyalkanoates

Members of the *Bacillus* spp. have been recognized as polyhydroxyalkanoates (PHAs) producers for many years, starting in 1926 as Maurice Lemoigne discovered poly‐β‐hydroxybutyrate (PHB) in *P. megaterium* (Mohapatra et al. 2017; Tsuge et al. 2015). *Bacillus* spp. serve as great models for PHA production due to their high growth rates during nutrient imbalance and with low‐cost substrates as carbon and nitrogen sources (Martínez‐Herrera et al. 2023; Mohapatra et al. 2017). PHAs are biodegradable polyesters synthesized by microorganisms under certain, often stressful conditions. They have garnered interest as a potential alternative to petroleum‐based plastics (Martínez‐Herrera et al. 2023). Given the rising concern over plastic pollution, PHAs present an opportunity to create a closed‐loop system of their use.

The initial screening test involved lipophilic staining of agar plates after bacterial cultivation with Sudan Black B (SB staining) (Muigano et al. 2023). After SB staining, bacteria containing PHAs exhibit dark granules (Figure 4A).

**FIGURE 4:**
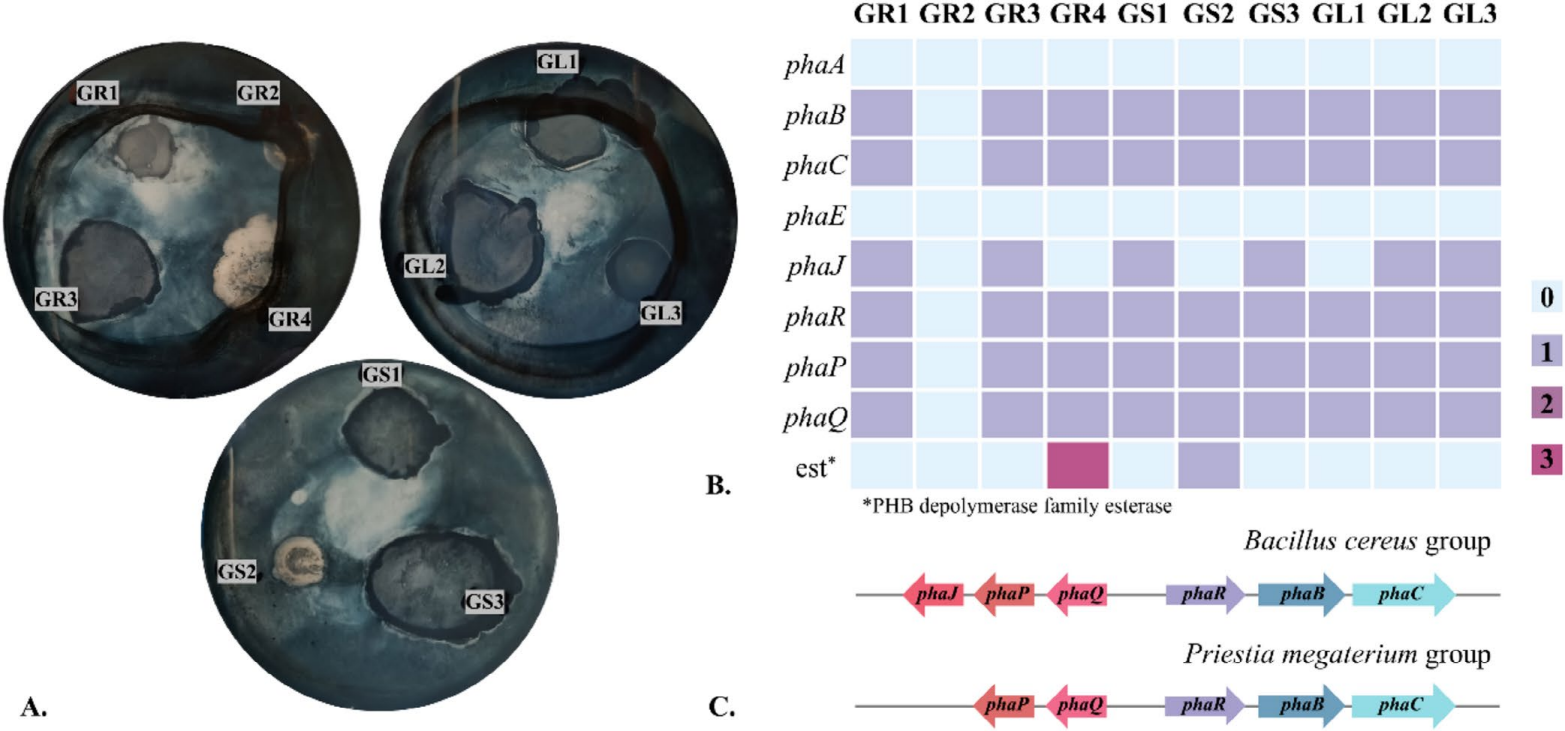
Polyhydroxyalkanoate (PHA)‐related characteristics of bacterial endophytes isolated from *Galium aparine* L. (A) Sudan Black staining results of the bacterial endophytes. (B) Detection of genes involved in PHA metabolism. (C) Organization of *pha* gene clusters in the analysed strains.

The ability to produce PHA is encoded within the genomes of nearly all bacteria associated with *G. aparine* L., except for *P. frigoritolerans* GR2 (Figure 4B). The bacteria producing PHA contain genes responsible for the process: *phaC* (encoding for poly(R)‐hydroxyalkanoic acid synthase subunit), *phaR* (polyhydroxyalkanoic acid synthase subunit), *phaP* (polyhydroxyalkanoic acid inclusion protein) and *phaQ* (poly‐beta‐hydroxybutyrate‐responsive repressor) (Figure 4C) (Kihara et al. 2017). Only three strains have been identified as non‐PHA producers. Since no genes linked to PHA or PHB production were found in *P. frigoritolerans* GR2, the absence of production is justified. The other non‐producing strains, which are *P. megaterium* GR4 and *Priestia* sp. GS2, have the *pha* genes listed above, but also possess genes encoding PHB depolymerase‐family esterase. Notably, *P. megaterium* GR4 has a set of three. That can make a sole difference in the type of produced PHA. Depolymerases metabolize PHA, thus, are responsible for their molecular weight decrease (Tsuge et al. 2015). Consequently, low‐molecular‐weight PHA may not get stained, meaning GR2, GR4 and GS2 strains can produce undetectable PHA by that assay.

Stress conditions play a crucial role in the production of PHA by bacteria. Therefore, the search for highly efficient strains should focus on environments exposed to stress, such as extreme habitats. For instance, Muigano et al. (2023) identified high‐yield PHA producer strains from soil, sediment, and water from the haloalkaliphilic lakes, including the best one, *Bacillus* sp. JSM‐1684023, which was further optimized for production (Muigano et al. 2023). Another promising approach to search for novel PHA producers is also bacteria associated with plants, as they are subjected to fluctuations in their habitat. *Bacillus aerophilus* A2, a peppermint endophyte, was able to produce up to 5.07 g/L of PHA on a soapstock, and slightly less (4.63 g/L) on beet molasse, another low‐cost substrate (Ignatova et al. 2023). Using such substrates significantly reduces the costs of PHA production, which can be more expensive than the production of petroleum‐based plastics. Moreover, optimizing PHA production can further enhance the yield. After detailed optimization of carbon and nitrogen sources, initial pH, and incubation period using response surface methodology (RSM), Das et al. (2022) achieved a 1.6‐fold increase in the PHA yield up to 12.6 g/L produced by *B. cereus* RCL02 isolated from the oil‐yielding plant *Ricinus communis* L. (Das et al. 2022).

#### 2.5.2 Other Metabolites Found in the Genomes

None of the tested extracts derived from lyophilized supernatant of isolates exhibited antibacterial and/or antifungal activities towards *Escherichia coli* , *Pseudomonas aureginosa*, *B. licheniformis* , *Mucor racemosus*, or *F. oxysporum*, despite the presence of BGCs responsible for the synthesis of known metabolites with this function. This outcome suggests that these bacteria do not produce such compounds under applied conditions, or in very low, undetectable levels.

However, we previously reported that the cluster displaying 87% similarity to the koranimine biosynthetic gene cluster is located in the genome of *P. frigoritolerans* GR2, but the capability of koranimine production by this strain was not experimentally verified (Rutkowska et al. 2025). In the present study, the UPLC‐MS analysis of ethyl acetate extract from the TSB medium‐based culture of *P. frigoritolerans* GR2 revealed the presence of a metabolite exhibiting the *m/z* of 804.5023 under positive ionization mode (ESI^+^) (Figure S1A). This value agreed with the theoretical *m/z* calculated for the [M + H]^+^ ion of koranimine, equal to *m/z* = 804.5018, with the relative mass error of 0.6 ppm. To provide a confirmation of koranimine identity, the MS/MS analysis was performed. The recorded mass spectrum (Figure S1B) included the peaks at the *m/z* values of 786.4949, 703.4534, 640.4066, 574.3611, 556.3470, 541.3377, 475.2953, 394.2726, 360.2312, 328.2259, and 281.1847, in agreement with the previous reports on koranimine fragmentation patterns (Evans et al. 2011; Montecillo and Bae 2022).

Evans et al. (2011) discovered koranimine using a ‘proteome‐first’ strategy by scanning bacterial proteomes for expressed gene clusters (Evans et al. 2011). Koranimine's structure has been determined by multistage tandem mass spectrometry (MS^n^), feeding studies with stable isotope tracers, ICP‐MS, nuclear magnetic resonance (NMR), and in vitro enzyme reconstitution. Koranimine was not found to inhibit the growth of *E. coli* , *S. aureus* , *E. faecalis* , *K. pneumonia*, or *S. cerevisiae* . The second study of the biological activity of koranimine revealed potential nematicidal activity (Montecillo and Bae 2022). Montecillo and Bae (2022) combined both genomic and metabolomic approaches to investigate the biosynthetic potential of endophytic *P. frigoritolerans* BE93, which was found before to exhibit nematocidal activity against *Bursaphelenchus xylophilus* causing pine wilt disease (PWD). *In silico* molecular docking revealed that koranimine can bind to the allosteric site of the invertebrate glutamate‐gated chloride channel (GluCl) in the same place as ivermectin, the natural target of the site.

## 3 Conclusions

This study demonstrates that *G. aparine* L. harbours diverse endophytic Bacillaceae exhibiting significant abiotic stress tolerance, traits that promote plant growth, and metabolic versatility. All isolates were capable of producing phytohormone IAA, ammonia, and hydrolytic enzymes, thereby directly supporting plant development and facilitating nutrient acquisition. Among them, *P. megaterium* GR4 emerged as a particularly promising strain due to its strong biosurfactant activity, ligninolytic capacity, and ability to produce pigments. *P. frigoritolerans* GR2 showed high potential through its efficient IAA production, nitrogen fixation, and confirmed synthesis of koranimine, while *B. wiedmannii GL3* distinguished itself by producing pulcherrimin, a pigment with antimicrobial and iron‐chelating properties. These features not only enable successful plant colonization but also enhance host resilience under adverse environmental conditions. Collectively, our findings underscore the significance of *G. aparine* ‐associated endophytes as essential partners in promoting plant health and fitness, presenting valuable opportunities for sustainable agriculture, biocontrol, and biotechnological applications.

## 4 Experimental Procedures

### 4.1 Biological Material

The bacterial endophytes were isolated from surface‐sterilized healthy tissues of medicinal plant *G. aparine* L. and preserved as pure cultures in 25% glycerol (v/v) at −80°C. In our previous work, bacteria were taxonomically identified based on their whole genome sequences. Their genomes were assembled, mined for genes involved in plant growth regulation and the production of bioactive compounds (Rutkowska et al. 2025).

### 4.2 In Vitro Assay for Stress Tolerance in Response to Various Temperatures, Drought and Salinity

All of the identified isolates were inoculated on LB agar medium with 20 μL of a bacterial suspension adjusted to an OD_660_ of 0.6 and for 48 h and incubated at 20°C–55°C in 48 h to assess their growth limits. The salt tolerance was evaluated by cultivation of isolates in LB medium (48 h, 30°C, 120 rpm) containing 0.5%–15% of NaCl (Gupta and Pandey 2019). To evaluate the drought tolerance, all isolates were grown in LB medium (48 h, 30°C, 120 rpm) supplemented with polyethylene glycol (PEG 8000) at various concentrations (0%, 10%, and 20%) to imitate water stress conditions (Ali et al. 2013; Gupta and Pandey 2019). The inoculum volume corresponded to 5% of the total cultivation volume and was prepared from the same standardized bacterial suspension (OD_660_ = 0.6). All experiments were performed in triplicate.

### 4.3 Nitrogen Fixation Ability

Isolates were screened for nitrogen‐fixing abilities by spot inoculation (20 μL of a bacterial suspension adjusted to an OD_660_ of 0.6) on the Jensen's agar medium (sucrose, 20 g; K_2_PO_4_, 1 g; MgSO_4_, 0.5 g; NaCl, 0.5 g; Fe_2_SO_4_, 0.1 g; Na_2_MoO_4_·2H_2_O, 0.005 g; CaCO_3_, 2 g; agar, 15 g; bromothymol blue, 0.5 g; dH_2_O, 1 L; pH 7.2 ± 0.2) and incubation at 30°C for 7 days (Abdel‐Hamid et al. 2021). The colour change of the medium around colonies from dark blue to green/yellow indicates positive nitrogen‐fixing ability. All assays were performed in triplicate.

### 4.4 Phosphate and Zinc Solubilization Abilities

Isolates were screened for phosphate and zinc solubilizing abilities by spot inoculation (20 μL of a bacterial suspension adjusted to an OD_660_ of 0.6) on the Pikovskaya agar medium (glucose, 10 g; Ca_3_(PO_4_)_2_, 5 g; (NH_4_)_2_SO_4_, 0.5 g; NaCl, 0.2 g; MgSO_4_·7H_2_O, 0.1 g; KCl, 0.2 g; FeSO_4_·7H_2_O, 0.002 g; yeast extract, 0.5 g; MnSO_4_ ·2H_2_O, 0.002 g; agar, 20 g; dH_2_O, 1 L; pH 7.0 ± 0.2) and Tris‐minimal agar medium (Tris–HCl, 6.06 g; NaCl, 4.68 g; KCl, 1.49 g; NH_4_Cl, 1.07 g; Na_2_SO_4_, 0.43 g; MgCl_2_ · 2H_2_O, 0.2 g; CaCl_2_ · 2H_2_O, 30 mg; ZnSO_4_, 1 g; agar, 15 g; dH_2_O, 1 L; pH 7.0 ± 0.2), respectively (Abdel‐Hamid et al. 2021; Gupta and Pandey 2019). The observation of clear zones formed around colonies after 7 days of incubation at 30°C was considered a positive result. All assays were performed in triplicate.

### 4.5 Ammonia Production

Each isolate was inoculated (with inoculum corresponding to 5% of the cultivation volume with OD_660_ of 0.6) into glass tubes containing 10 mL of peptone water (peptone, 10 g; NaCl, 5 g; dH_2_O, 1 L; pH 7.0 ± 0.2) and incubated at 33°C for 72 h. Then, 0.5 mL of Nessler's reagent was added to the culture broth. The development of yellow colour indicates ammonia presence (Abdel‐Hamid et al. 2021). All assays were performed in triplicate.

### 4.6 IAA Production

The production of indole‐3‐acetic acid (IAA) was assessed by cultivating isolated strains on LB medium supplemented with 0.2% (w/v) L‐tryptophan (28°C, 120 rpm, dark). The inoculum corresponded to 5% of the cultivation volume with OD_660_ of 0.6. Each culture supernatant was obtained by centrifugation (11,000 rpm, 10 min, 4°C) and then used for the estimation of IAA content. The supernatant was mixed with Salkowski reagent (0.5 M FeCl_3_·6H_2_O in 35% HClO_4_) in a 1:2 ratio and incubated in the dark for 30 min at room temperature (23°C), followed by absorbance measurement at 530 nm (Glickmann and Dessaux 1995; Suliasih and Widawati 2020). To determine the amount of auxins produced, a standard curve with known concentrations (0–100 μg/mL) of commercial IAA (Sigma‐Aldrich) was used. All experiments were conducted in triplicate, and the results are shown as the mean ± standard deviation (SD).

### 4.7 Extracellular Hydrolytic Enzymes Production

The production of extracellular hydrolytic enzymes was screened by inoculating isolates (using inoculating loop of 1 μL) in LB agar medium with specific additives, depending on the tested enzymatic activity. The LB agar media with an additive without inoculation was used as a control in each test. Enzymatic activities were evaluated using a qualitative plate‐based screening approach. All assays were performed in triplicate (three independent biological replicates). The presence of extracellular hydrolytic activity was assessed based on the formation of visible substrate degradation zones around bacterial colonies after 48 h of incubation at 30°C. Results were categorized using a semi‐quantitative scoring system as follows: (−) no visible hydrolysis zone; (+) weak activity with a faint or small hydrolysis zone; (++) moderate activity with a clearly visible hydrolysis zone; (+++) strong activity with an intense and/or large hydrolysis zone relative to colony size.

For amylolytic activity, LB agar medium contained 2% of soluble starch. After the incubation period, the plates were flooded with 3% iodine for 15 min (Abdel‐Hamid et al. 2021). For cellulolytic, chitinolytic and chitosanolytic activities, LB agar medium contained 2% carboxymethylcellulose (CMC) and 2% of colloidal chitin (Kaczmarek et al. 2019). After the incubation period, the plates were flooded with 0.5% Congo Red solution for 15 min, then followed by flooding with 1 M NaCl for another 15 min (Oke et al. 2017). For proteolytic, lipolytic and esterolytic activities, LB agar medium was supplemented with 10% of skimmed milk, 2% olive oil and 2% of tributyrin, respectively (Abdelmoteleb et al. 2017; Amina et al. 2020; de Guzmán et al. 2008). For xylanolytic and pectinolytic activities, LB medium was amended with 1% of birchwood xylan and 1% of pectin, respectively (Nawawi et al. 2017). Then, after incubation, the plates were flooded with 3% iodine solution for 15 min. The positive results were indicated by the presence of a clear zone around bacterial growth. For laccase activity, 0.01% guaiacol and 0.35 mM CuSO_4_ were added to LB medium (Mandic et al. 2019). The positive result was a yellow‐brown colour around bacterial colonies.

### 4.8 Biosurfactants Production

Biosurfactants production by isolated strains was performed in standard LB medium (pH 7.0 ± 0.2) inoculated with inoculum corresponding to 5% of the cultivation volume with OD_660_ of 0.6. Endophytic strains were introduced into 100 mL Erlenmeyer flasks with 20 mL of the LB medium. Then, the culture was carried out for 8 days. Biosurfactant production was evaluated using emulsifying activity, emulsion index and oil displacement test. Shortly, the emulsifying activity of the supernatant was determined according to the method of Pearce and Kinsella (1978). The reaction mixture (3 mL of supernatant, 1 mL of diesel oil, 1 mL of 0.05 M phosphate buffer, pH 7) was homogenized for 30 s, then 0.1 mL of the sample was mixed with 1 mL of 0.1% SDS. Absorbance was measured at *λ* = 500 nm against distilled water. The medium without inoculation served as the negative control. The emulsion index was determined according to the method of Cooper and Goldenberg (1987). A mixture of 2 mL of hydrocarbon and 2 mL of aqueous solution of biosurfactant (1 mg/mL) was vigorously vortexed for 5 min and the emulsion stability was assessed after 24 h. IE24 was calculated as the ratio of the emulsion layer height (*x*) to the total mixture height (*y*) × 100. The oil displacement test was assessed according to the method of Pornsunthorntawee et al. (2008). 10 μL of biosurfactant solution was applied to 50 μL of used engine oil placed in 50 mL of water in a 10 cm diameter Petri dish. After 5 min of incubation at room temperature, the diameter of the clearing zone was assessed. The presence of carbohydrate groups was analysed by the method of Dubois et al. (1956). To 1 mL of biosurfactant solution (10 mg/mL) was added 0.5 mL of 5% phenol and 2.5 mL of concentrated H_2_SO₄, incubated for 30 min at room temperature, and then the absorbance was measured at λ = 490 nm. The presence of peptide bonds was determined by the method of Bradford (1976). To 1 mL of biosurfactant solution was added 1 mL of 1 M NaOH and heated in a boiling water bath for 10 min. After cooling, 50 μL of the sample was mixed with 1.5 mL of Bradford reagent and the absorbance was measured at λ = 590 nm against the reagent sample with distilled water.

### 4.9 PHA Production

The isolates were assessed for the production of polyhydroxyalkanoates (PHA). After their cultivation on LB agar medium (72 h, 30°C) with 1 μL of biomass using an inoculation loop, plates were stained with 0.05% ethanolic solution of Sudan Black B for 30 min, then rinsed with pure ethanol to remove excess stain (Bhuwal et al. 2013). Colonies of PHA‐positive isolates incorporated the stain and appeared bluish black.

Genes associated with PHA production were found using PGAP annotation available for each genome at NCBI GenBank Database. All genomes of *G. aparine* L.'s isolates were deposited under BioProject no. PRJNA 1068863. The regions in proximity to found single genes (f.e. *phaQ* or *phaR*) were further investigated in detail using blastn and blastp tools to find the rest of genes in the predicted cluster, as PGAP annotation was not sufficient. All experiments were performed in triplicate.

### 4.10 Pigment Production

Based on analysis of present gene clusters and the colour of their colonies on standard LB agar medium, some strains were further tested. Strains *Priestia* sp. GS2 and *P. megaterium* GR4 were tested for carotenoid production and strain *B. wiedmannii* GL3 was tested for pulcherriminic acid production (Rutkowska et al. 2025). For carotenoid production, strains *Priestia* sp. GS2 and *P. megaterium* GR4 were inoculated in NB medium (beef extract, 1 g; peptone, 5 g; yeast extract, 2 g; NaCl, 5 g; dH_2_O, 1 L; pH 6.8 ± 0.2) with inoculum corresponding to 5% of the cultivation volume with OD_660_ of 0.6 and cultivated for 72 h (30°C, 150 rpm), then centrifuged (10,000 rpm, 15 min, 4°C). Pigmented biomass was mixed with methanol in a ratio 1:2 (w/v), vortexed for 1 min and incubated in a water bath at 60°C for 15 min as according to Hagaggi and Abdul‐Raouf (2023). Yellowish supernatant was centrifuged and lyophilized. For pulcherrimin production, *B. wiedmannii* GL3 was first tested on TSB agar medium (casein peptone, 17 g; soya peptone, 3 g; glucose, 2.5 g; NaCl, 5 g; K_2_HPO_4_, 2.5 g; agar, 20 g; dH_2_O, 1 L; pH 7.3 ± 0.2) with various FeCl_3_ concentrations, inoculated with 1 μL of biomass using an inoculation loop. All experiments were performed in triplicate.

### 4.11 Antimicrobial Activity

All of the isolates were cultivated in LB medium (30°C, 120 rpm, 48 h) inoculated with inoculum corresponding to 5% of the cultivation volume with OD_660_ of 0.6. Culture supernatants were obtained by centrifugation (11,000 rpm, 4°C, 10 min), then lyophilized. Lyophilized supernatants (LSs) were dissolved in sterile water, diluted and filter‐sterilized. To evaluate the antimicrobial activity, the disk diffusion method was used (Marchut‐Mikołajczyk et al. 2023). The LSs were placed onto 1‐mm‐diameter sterile filter paper disc. Representative bacteria ( *E. coli* DH5α, *P. aureginosa* ATCC 1544, *B. licheniformis* NCIMB 8059) were inoculated on LB agar, and fungi (*M. racemosus* CH52, *F. oxysporum* 1101) on Czapek‐Dox for (sucrose, 30 g; K_2_HPO_4_, 1 g; MgSO_4_, 0.5 g; KCl, 0.5 g; FeSO_4_, 0.01 g; agar, 30 g; dH_2_O, 1 L, pH 7.0 ± 0.2). All strains are from the collection of Institute of Molecular and Industrial Biotechnology of Lodz University of Technology. Then on top of those plates, sterile filter paper discs containing 2.5, 5, 10 or 20 mg/mL of each LSs were deposited. Plates were incubated at 30°C for 24 h (for bacteria) or 72 h (for fungi) and examined for the formation of halo zones around colonies. The positive control consisted of filter paper discs laden with 10 μg of gentamicin (Oxoid Gentamicin Antimicrobial Susceptibility Discs, Thermo Scientific). All experiments were performed in triplicate.

### 4.12 UPLC‐MS Analysis

After 48 h of cultivation (30°C, 180 rpm) of each isolate in 100 mL of TSB medium (using standardized inoculum as stated previously), culture broth was sonicated (time 4 min, temperature 8°C, pulse 30 s). After that, supernatants were collected by centrifugation (11,000 rpm, 10 min, 4°C). Only strain GL3 was cultivated in minimal ‘A’ medium (glucose, 2 g; yeast extract, 2 g; Na_2_HPO_4_, 1.5 g; NH_4_Cl, 2.5 g; agar, 25 g; dH_2_O, 1 L; pH 6.5 ± 0.2), as extraction of culture supernatant from TSB medium with ethyl acetate, chloroform or acetone resulted in the formation of a semisolid layer. Then, supernatants were acidified to pH 2.5 with 6 M HCl and extracted three times with an equal volume of ethyl acetate (Patel et al. 2016). The ethyl acetate phases were separated and concentrated using a rotary evaporator. Extracts were subjected to UPLC‐MS analysis with the use of AQUITY‐UPLC apparatus coupled with the SYNAPT G2 mass spectrometer (WATERS, USA), and screened for the presence of bioactive compounds synthesized by proteins encoded within BGCs (Rutkowska et al. 2025). The chromatographic column BEH Shield RP18 2.1 mm × 100 mm × 1.7 μm (WATERS, USA) was used over the course of UPLC‐MS analysis. Water (A) and acetonitrile (B), both acidified with formic acid (0.1% v/v), were employed as eluents. The following A:B gradient was applied: initial 100:0; 5.00 min—80:20; 11.00 min—70:30; 15 min—60:40; 25.00 min—40:60, 35.00 min—20:80; 45.00 min—0:100. During the analysis, the temperature values of the samples and the column were equal to 12°C and 40°C, respectively, while the flow rate through the column was kept at 0.2 mL min^−1^. The MS voltage values were set to: 40 V (sampling cone), 4 V (extraction cone), and 3 kV (capillary voltage). The temperature of the MS source and desolvation was equal to 120°C and 200°C, respectively. The desolvation gas flow rate was kept at 500 L h^−1^. The solution of leucine enkephalin was used as a lock mass reference. The 5 mM sodium formate solution in 90:10 2‐propanol/water, prepared according to the MS manufacturer's instructions, was employed for the calibration of the SYNAPT G2 (WATERS, USA) instrument prior to the analysis of the samples. The acquisition of data and the automatic peak detection was performed with the use of MassLynx software (WATERS, USA). The MS/MS analysis, with the parent ion set to *m/z* = 804.5023, was carried out to confirm the identity of koranimine. All analyses were performed under the positive electrospray ionization mode (ESI^+^).

## Author Contributions

**Natalia Rutkowska:** conceptualization, methodology, data curation, investigation, formal analysis, visualization, writing – original draft, project administration. **Piotr Drożdżyński:** conceptualization, methodology, investigation, formal analysis, writing – original draft. **Tomasz Boruta:** methodology, software, investigation, formal analysis, writing – original draft, resources. **Bartosz Sekuła:** supervision, writing – review and editing. **Olga Marchut‐Mikołajczyk:** conceptualization, writing – review and editing, resources, supervision.

## Funding

This work was supported by Narodowe Centrum Nauki (SONATA‐BIS 2025/58/E/NZ8/00097).

## Conflicts of Interest

The authors declare no conflicts of interest. All authors have read and approved the manuscript. This research did not receive any specific grant from funding agencies in the public, commercial, or not‐for‐profit sectors.

## Supporting information


**Table S1:** IAA concentration (μg/ml) at different hours of cultivation in LB medium supplemented with 0.2% (w/v) L‐Trp
**Figure S1:** (A) The extracted ion chromatogram (EIC) corresponding to *m/z* = 804.502, recorded for the ethyl acetate extract from the TSB medium‐based culture of *P. frigoritolerans* GR2 and (B) The ESI^+^ MS/MS spectrum obtained for the parent ion at *m/z* = 804.5023.

## Data Availability

The data that supports the findings of this study are available in the Supporting Information of this article. There were no human or animal subjects.
