# Supplementary material for: Bacillaceae Within Galium aparine L.: A Plant‐Associated Treasure of Biosynthetic Diversity and Growth‐Promoting Potential
Source: Environ Microbiol Rep. 2026 Oct 23;18(5):e70447. doi: 10.1111/1758-2229.70447 (PMC13694211; doi:10.1111/1758-2229.70447)
Supplement: Supplementary file 1 [file EMI4-18-e70447-s001.docx]

**Supporting Information**

**Table S1.** IAA concentration (µg/ml) at different hours of cultivation in LB medium supplemented with 0.2% (w/v) L-Trp

| **Strain** | **48 h** | **72 h** | **96 h** | **120 h** |
| --- | --- | --- | --- | --- |
| **GR1** | 3.75 ± 0.20 | 5.18 ± 0.23 | 10.12 ± 0.29 | 9.98 ± 0.56 |
| **GR2** | 11.00 ± 0.66 | 19.52 ± 0.33 | 24.33 ± 1.31 | 24.15 ± 0.25 |
| **GR3** | 4.37 ± 0.69 | 6.40 ± 1.01 | 11.53 ± 0.30 | 13.18 ± 0.31 |
| **GR4** | 9.83 ± 1.06 | 9.69 ± 0.11 | 14.18 ± 0.72 | 15.55 ± 0.11 |
| **GS1** | 0.47 ± 0.27 | 7.67 ± 0.65 | 11.33 ± 0.05 | 12.03 ± 0.23 |
| **GS2** | 0.83 ± 0.13 | 5.60 ± 0.33 | 9.56 ± 0.35 | 10.67 ± 0.31 |
| **GS3** | 2.61 ± 0.19 | 7.48 ± 1.19 | 10.65 ± 1.20 | 12.51 ± 1.54 |
| **GL1** | 3.35 ± 0.93 | 17.48 ± 1.10 | 14.54 ± 0.34 | 15.59 ± 0.26 |
| **GL2** | 2.31 ± 0.23 | 7.01 ± 0.36 | 10.38 ± 0.21 | 11.82 ± 0.19 |
| **GL3** | 2.13 ± 0.16 | 5.92 ± 0.09 | 9.96 ± 0.69 | 12.16 ± 0.51 |

**Figure S1. A.** The extracted ion chromatogram (EIC) corresponding to *m/z* = 804.502, recorded for the ethyl acetate extract from the TSB medium-based culture of *P. frigoritolerans* GR2, **B.** The ESI^+^ MS/MS spectrum obtained for the parent ion at *m/z* = 804.5023
